# The roads to Universal Health Coverage: Manifest destiny or Sisyphean pursuit?

**DOI:** 10.7189/jogh.11.03096

**Published:** 2021-11-20

**Authors:** Olusoji Adeyi, Mickey Chopra, Allyala Nandakumar

**Affiliations:** 1 Resilient Health Systems. Washington, D.C., USA; 2 The World Bank. Washington, D.C., USA; 3 Brandeis University, Waltham and Office of the United States Global AIDS Coordinator, Washington, D.C., USA

This is the age of Universal Health Coverage (UHC). The ideal of ensuring access to key promotive, curative and rehabilitative health interventions for all at an affordable cost underlies most targets in the Sustainable Development Goal 3. It arises as part of broader reform efforts in wealthier countries to invest more in health systems, rather than individual programs and facilities, to address health needs of aging populations, high and increasing incidence of NCDs and escalating costs. For lower income countries, UHC is seen as a way of reaching all the people with essential health services without financial hardship; overcoming entrenched vertical disease programs and being stuck in providing a relatively narrow set of interventions that are now limiting further progress. The United Nations General Assembly in 2019 adopted a resolution that included a sweeping recommitment “to achieve universal health coverage by 2030” [1], in what the UN Secretary General called “the most comprehensive agreement ever reached on global health – a vision for Universal Health Coverage by 2030” [2].

The reality for millions of people across the world is that UHC remains an elusive goal despite global proclamations and resolutions, and despite statements of intention by policy makers at the country level. The latest Global Monitoring Report for UHC makes for sobering reading: the pace of progress in coverage of services has slowed since 2010; financial protection is deteriorating instead of improving, with more people suffering hardships as a result of large out-of-pocket spending at the point of service delivery; and inequalities are widening. [3] Without significant progress that is clear to, and felt by, the underserved and the under-privileged, disillusionment could seep into populist discourse as the blame for the perceived lack of progress is placed at the door of immigrants and the “undeserving” poor.

Some analysts have identified systematic and structural challenges within the health sector including transforming the acute-care and “illness treatment” orientation of care models, reversing often top-heavy structures of the service delivery systems, and reducing service fragmentation and siloed management among facilities and service levels [4-6]. Such factors have indeed played a role in the lack of progress but, according to the papers in this series, they also obscure more profound and important mismatches between expectations and reality that will continue to stifle efforts to achieve UHC unless they are more fully described and addressed.

## TENSIONS

This series highlights three critical tensions between expectations/assumptions and reality:

1. Political premise: Political dividends of UHC can overcome implementation challenges and competing demands from different constituencies.
2. Financing: Increased public financing will help reduce the impoverishing out-of-pocket expenditures at the point of service delivery and achieve economies of scale and other efficiencies.
3. Technology and service delivery: Automation, adaptation of proliferations of mobile app, application of data science to supply chain, and use of remote-access technologies for diagnosis and treatment support will enhance the overall performance of service delivery systems.

### Political premise

Historically, the promise of UHC was used by the likes of Germany, Bevan in the UK and the ruling Liberal Democrats in Japan to make the provision of more organized and widespread health care to ameliorate the increasing social disruption caused by industrial revolution, war, and reconstruction respectively. So, it is not surprising to see many politicians embracing UHC as a potential vote winner (for example, the 2018 mid-term elections in the USA). In high-income settings, access to high quality services became one emollient against the rising extreme inequalities and insecurities battering the fragile social contract in many countries. In low- and middle-income settings, the immediate impetus in adopting UHC was the growing realization of the limitation of more selective, disease specific approaches that dominated global health during the period of widespread structural adjustment and the subsequent Washington Consensus.

### Political realities

However, the seemingly pragmatic approach of focusing upon relatively narrow and highly cost-effective health interventions is crashing against a rising tide of expectations from populations for whom access to quality health care and better health is an increasing expectation. What one party views as pragmatic could be seen by other parties as an abdication of responsibility, especially when UHC is framed not as a privilege but as a human right. The hitherto understated dissonance at the core of the drive for UHC is, to some extent, a reprise of debates about Primary Health Care and Health For All in the wake of the 1978 Alma Ata Declaration [7]. The wide gap between global declarations and ground realities prompted a technology-and-delivery based movement for selectivity and quick wins in the form of “Selective Primary Health Care” [8], a proposition that was subsequently branded as a “counter-revolution” [9]. Clearly, the goals of “Health for All by the Year 2000” were not realized. That failure raises the stakes for the current aspirations for UHC by 2030.

**Figure:**
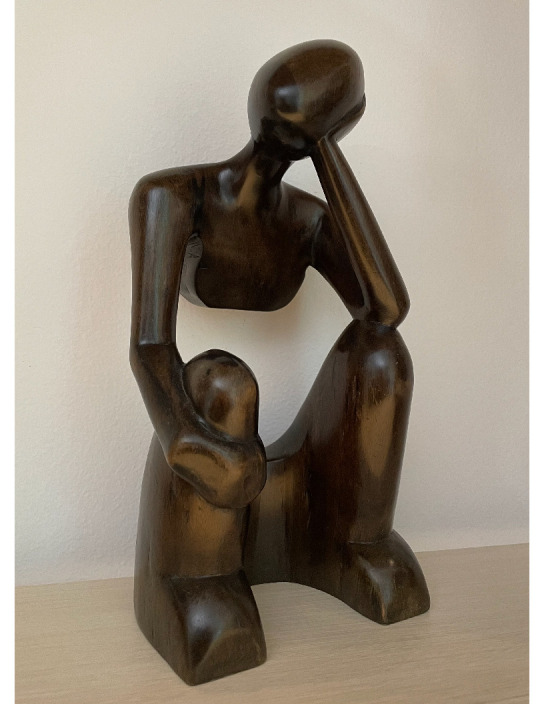
Photo: A village artist’s rendition of the thinker (Olusoji Adeyi, personal collection).

Within countries, there are very positive signs of works in progress. The Government of Kenya has committed to UHC [10] and the country is working through a pilot and multiple institutional reform amidst its still recent political devolution of powers to the counties. Elsewhere [11], political transitions from one government to another may delay needed changes, as appears to be the case in Ghana, where the recommendations to improve its National Health Insurance System have not gained any noticeable traction, at least publicly. In South Africa, a major challenge consists of major reforms of how current funds are pooled and services are purchased, which requires huge political will for a major restructuring of the responsibilities in the health sector. The case for public financing as a necessity for realizing UHC is eloquently made by two authors, who also contend that equity is intrinsic is to the very notion of UHC; in a tour de force, they draw explicit attention to the centrality of redistribution in the health financing transition to UHC and the necessary policy choices [12].

### Financing expectations

Public financing is the largest single source of funding in most high- income countries (HICs) and middle-income countries (LICs) and it has the potential to be an effective and efficient actor in the health sector. This is not the case in most low-income countries, especially those with high donor dependence. In these countries public financing is a smaller portion of the financing landscape. In Sub-Saharan Africa in over a dozen countries donor spending on health exceeds public spending on health and as donor spending has increased it has partly crowded out public spending on health [13]. Social health insurance schemes tend to cover primarily the formal sector, and these too are under financial stress with the pressure to increase benefits, expand the use of private providers and emergence of new technologies. Fuch’s [14] conditions to achieving UHC – subsidization and compulsion, the public sector necessarily provides the mechanism for redistributing resources to enable subsidization and the authority to enforce mandatory health care or insurance coverage. Two points merit attention in this context. First, these public-sector oriented solutions mask the importance of increasing public sector efficiency. Second, whether such improvements in efficiency come from within the public sector itself, or via integration or leveraging private sector actors, should be matters for empirical work, not ideological preferences.

Many mechanisms that enhance efficiency and accountability mimic market behaviors, particularly through contracting private providers and reforming purchasing strategies to strengthen the incentive environment for both public and private providers to deliver quality, accessible, affordable, and accountable care. Furthermore, the economics of health care was now purported to be in favor of providing universal coverage. The success of large-scale health programs such as PEPFAR, GFATM and GAVI in bringing down the costs of essential medicines, vaccines and delivery systems showed how economies of scale could be realized. The revitalization of PHC was another strategy shown to reduce costs and an essential component of UHC.

### Financing realities

The sticker shock for the cost of providing universal health coverage, the latest UHC Global Monitoring Report estimates a shortfall of at least 175 billion dollars just in low and middle income countries, and the realization that out of pocket expenditure, currently estimated at almost half a trillion dollars, is not decreasing, reflects the reality that any efficiency gains are being overwhelmed with the surge in pent up demand for health care. This in turn has opened a vigorous debate regarding the role of public and private financing. It is well established that there is a positive correlation between the income of countries and the proportion of gross domestic product spent on health. However, as highly donor dependent countries transition from low income to low middle-income status one observes a pulling back from donors, the government not stepping in to fill the gap and an increase in out-of-pocket spending. [15] External financiers’ requirements of country contributions mean that governments have less discretion on how to spend their health dollars. At the same time a worrying trend is the increase in debt to GDP ratios in many countries in Sub-Saharan Africa that have embraced UHC as a key policy objective. Despite the inherently compelling proposition of self-reliance through domestic resource mobilization, it is becoming increasingly clear that a publicly financed UHC model is coming face-to-face with the fiscal realities facing nations.

There has been a widespread failure to recognize that health care is made up of public, merit and private goods. Public goods such as defense and social justice are by their very nature are indivisible and the consumption by one individual does not decrease the availability for another individual. There appears to be consensus that public goods will need to be publicly financed. Merit goods are those where there is a benefit to both the individual as well as society. There is very little written about or consensus on how to finance and provide merit goods. Private goods are those where the benefit accrues primarily to the consumer and here markets do well in meeting consumer demand. Beyond these facts, there isn't a consensus on what to do with merit and private goods, and no consensus on the “right” combination of public and private approaches to the financing, management, and delivery of services in country-wide systems.

Some commentators point out that as incomes rise the revealed preference of individuals and households will change. People will tend to have fewer children and invest more them in creating the human capital that can then be deployed to optimize income and well-being (Musgrave’s human capital theory) [16]. This will mean that households will tend to use market-based solutions, if in their view, these are better than the publicly financed and provided services. This is something that neither governments nor international organizations can control. Markets will respond to these demands. Failure to recognize these realities will only lead to increased fragmentation and difficulties in achieving economies of scale.

Others have chafed against the greater role given to private financing and argue that since political leaders have formally endorsed UHC as a desired goal of public policy, the challenge becomes how to deploy both public and private means to achieve those public policy goals as equitably and efficiently as possible. There is no evidence to support a proposition that privately financed, privately managed, and privately delivered health care systems are superior to others in terms of achieving and maintaining equitable and efficient UHC. Market failures are common and serious in health, and there are myths about the capacity of the private sector alone to achieve efficiency and control costs [17]. Notions premised on consistently rational behaviors in health-seeking and utilization behaviors stand on shaky grounds [18]. The recognition of equity and merit goods, while sound, was in the past drowned out by a concurrent push for user fees [19]. At the same time, there are widespread and sometimes massive government failures, manifested in various degrees of rent seeking and elite capture. Thus, the optimum combination of public and private sector engagement must be context-specific. Given the historical injustice and continuing legacy of the apartheid-era health system in South Africa, and the inequities of the private health insurance market in that country, it is not surprising that many stakeholders there favor a heavy public sector hand if they perceive that as a potentially restorative prospect. The appropriate mix of government and private engagement in that country, as elsewhere, is a matter for empirical work with attention to the political economy of UHC [20].

### Technology and service delivery expectations

Global health experts added further gasoline in touting the transformative potential of technology and big data to increase the efficiency and effectiveness of health services. A common example is the electronic health record, which has been shown to improve clinical decision support, registries, team care, care transitions, personal health records, telehealth technologies, and measurement [21]. When these key factors function smoothly in a health care setting, both providers and patients experience a more coordinated care pathway. Providers across different levels can communicate in real-time and easily access current and new patients’ health information in one place. The contemporary experience in Ghana highlights the potential of technology to improve systemic efficiency; the Ghanaian NHIA has adopted Electronic Claims Processing as a mechanism to manage logistical challenges associated with paper claims management, with a view to boosting efficiency in claims processing, improve transparency to providers, and provide credible claims data for analysis. In India, it is recognized that an interoperable IT system would help to support a continuum of care approach with seamless integration of preventive, primary, secondary, tertiary and follow-up care. Data systems interoperability and data sharing may be necessary in Indonesia too, to increase the chances of achieving and sustaining UHC.

### Technology and service delivery realities

Technology, especially digital technology, is already being deployed to enable progress along the three dimensions of UHC, ie, coverage, quality, and financial protection. Experiences and insights from the field show opportunities for even more progress [22].

Many of the vertical programs have created parallel delivery systems. Integrating these parallel systems would enable economies of scope, but doing so will be difficult and we expect to see variations in the extent of integration. [23] In many countries the private for profit and not for profit sectors are the predominant provider of outpatient services (we need to have some illustrative examples for this). However, this sector remains fragmented, unregulated and of uneven quality. The UHC discussion must recognize this reality and ensure that there is a systematic effort to “formalize” this sector and integrate it into the mainstream of service delivery.

This fragmentation, weak capacity, and stewardship are also stymying the potential impact of new technologies, big data analytics and use of artificial intelligence for health. Examples include: (a) predictive analytics is not yet widely applied in primary health care or trusted by LMIC governments; (b) few LMIC examples of ‘operationalized’ machine learning solutions that are

fully engineered, exist; (c) primary health care data are not yet widely available for machines to learn from; (d) virtually no predictive analytics that focuses on demand-side analytics exist – most health systems are designed using existing supply-side data; (e) almost all the current suite of mobile health / eHealth solutions are focused on digitizing data about health service delivery or provide in-service training and not on how such data are used to predict, pre-empt, personalize and transform health service delivery; (f) lack of regulation, or digital health strategies, by Governments; (g) lack of focus on the perils of artificial intelligence or risks to Governments; and (h) evidence of what works in this space is not well defined, particularly in the primary health care arena.

### Moving forward

Translating political commitments into reality is a central challenge of the UHC era. Policy makers, financiers, and those in positions to influence strategies and programs would benefit from the following considerations:

- Increase collaboration with existing health advocacy groups, civil society groups and organized labor organizations to further the use of UHC to ensure access to essential services.
- Smarter roadmaps towards UHC but also recognizing the path dependency nature of health systems and the dangers of promoting things like private health insurance etc.
- Keeping sight of the public health, social determinants agenda that can reap perhaps greater health benefits than just a focus upon health care.
- The positive takeaway from the country examples is that they are experimenting with different approaches both on the financing and service delivery side and are tailoring solutions that reflect the political and socio-economic realities on the ground. The analysis on the state of health financing and the case studies reflect the dominance of both public financing and publicly organized and regulated models of risk pooling in country strategies to progressively achieve UHC.
- To do this requires a willingness to be flexible and innovate. Many LMICs have dispensed with traditional models of organizing and pooling their resources. This has enabled mixed models that pool different sources of contributory and non-contributory revenues (such as from payroll taxes, general revenue financing, and sin taxes) and subsidies to include the hard to reach poor and non-poor informal sector.
- The countries discussed in the case studies have been promoting experimentation to find new ways to attract and work with the private sector. This includes thinking through new service delivery models to increase primary care services delivered by both the public and private sector.
